# Safety and Efficacy of Pressure-Enabled Thyroid Embolization: A Novel Approach for Symptomatic Thyroid Disease

**DOI:** 10.1210/jendso/bvaf117

**Published:** 2025-07-12

**Authors:** Sandra Gad, Nima Kokabi, Michael Mohnasky, Ralph P Tufano, Angela Boldo, Juan C Camacho

**Affiliations:** Division of Vascular & Interventional Radiology, Department of Radiology, University of North Carolina at Chapel Hill, Chapel Hill, NC 27514, USA; School of Medicine, St. George's University, West Indies, Grenada; Division of Vascular & Interventional Radiology, Department of Radiology, University of North Carolina at Chapel Hill, Chapel Hill, NC 27514, USA; School of Medicine, University of North Carolina at Chapel Hill, Chapel Hill, NC 27514, USA; Department of Interventional Radiology, Sarasota Memorial Hospital, Sarasota, FL 34239, USA; Department of Interventional Radiology, Sarasota Memorial Hospital, Sarasota, FL 34239, USA; Department of Interventional Radiology, Sarasota Memorial Hospital, Sarasota, FL 34239, USA; Radiology Associates of Florida, Sarasota, FL 34232, USA

**Keywords:** pressure-enabled drug delivery catheter, Thyroid Artery embolization, thyroid nodules, multinodular goiter (MNG), toxic goiter

## Abstract

**Context:**

To assess the safety and efficacy of pressure-enabled thyroid embolization (PED-TAE) for thyroid embolization.

**Objective:**

This work aimed to evaluate the safety, feasibility, and early efficacy of PED-TAE via the inferior thyroid arteries in patients with symptomatic thyroid disease.

**Methods:**

This retrospective cohort study took place at an academic outpatient clinic. Between May 2023 and July 2024, 22 patients underwent PED-TAE using a pressure-enabled drug delivery (PEDD) device. We retrospectively reviewed patient characteristics, procedure details, and adverse events. Patients were treated with PED-TAE predominantly via transradial access with 100 to 300 µm spheres. Ten patients (45%) underwent preplanned unilateral embolization and 12 (55%) bilateral embolization. Main outcome measures included successful embolization of the targeted thyroid lobe via an inferior thyroid artery only, as well as absence of nontarget embolization, target volume reduction, and normalization of thyroid function when appropriate.

**Results:**

Etiology included 11 (50%) multinodular goiters, 6 (27%) toxic nodules/goiters, 3 (14%) pre thyroidectomy, 1 (4%) Graves disease, and 1 (4%) solitary nodule. Technical and clinical success was achieved in all patients. Eighteen patients reported mild pain or discomfort, which resolved within 2 weeks. No neurovascular complications were reported. A total of 71% of patients with hyperthyroidism became euthyroid. Six-month follow-up data were available for 18 patients, in whom the mean gland volume decreased from 184.5 ± 141.4 mL pre procedure to 49.9 ± 33.7 mL (*P* < .05), with a mean reduction of 73%.

**Conclusion:**

PED-TAE is safe and feasible, with high euthyroid conversion rates and volume reduction. A multi-institutional study is planned to validate the findings.

Thyroid nodules are common, with a prevalence of 3% to 8% in the general population, and a higher prevalence is observed in women (5%) [1]. The majority of these nodules (95%) are benign and asymptomatic [1, 2]. However, benign thyroid nodules can become symptomatic due to compressive symptoms or autonomous function and, depending on the etiology, may rarely become malignant [3]. In addition, more than half of patients with benign thyroid nodules present as multinodular goiters (MNGs), defined as two or more nonpalpable or palpable nodules within an enlarged thyroid [4]. This patient population often presents with compressive symptoms, including dyspnea and dysphagia, mainly when the thyroid gland weighs more than 20 to 25 g or with a volume of more than 20 mL [4]. Traditionally, surgery has been the standard of care for the management of thyroid nodules [3]. Although thyroidectomy is safe, it can result in complications such as recurrent laryngeal nerve palsy (0%-2.1%), hypoparathyroidism (4%-25%), and permanent hypothyroidism that requires lifelong hormone therapy [5-8].

Advances in imaging, especially high-resolution ultrasound, have increased thyroid nodule detection, leading to the emergence of minimally invasive treatments like percutaneous ablation, radioactive iodine, and thyroid artery embolization (TAE). The aforementioned treatments are ideal for nonsurgical candidates or those wishing to preserve thyroid parenchyma. However, each has its own set of limitations—radioactive iodine may cause hypothyroidism, ablation is less effective for nodules larger than 20 mL, and TAE carries a risk of nontarget embolization [9].

TAE offers a treatment option for thyroid nodules beyond number and volume, provides symptom relief in hyperthyroid conditions, and serves as a preoperative or palliative intervention to reduce surgical morbidity and improve quality of life (QoL) [1-4]. In TAE, microspheres are delivered into thyroid arteries to obstruct blood flow and induce ischemic necrosis of the thyroid tissue.

Early studies demonstrated that TAE can significantly reduce nodule and goiter volume, improving QoL and hormonal status [5, 9]. However, TAE has not gained wide acceptance due to the potential risk of cerebrovascular accidents and nontarget embolization [10]. The arterial anatomy of the thyroid is complex, with the inferior thyroid artery branching from the subclavian and the superior thyroid artery branching from the external carotid [11] (Fig. 1). Traditionally, TAE requires catheterization of one or both superior thyroid arteries and one or both inferior thyroid arteries [12, 13]. This arterial anatomy presents the risk of nontarget embolization, especially when embolizing the superior thyroidal artery, because of the possible reflux of particles into the internal carotid artery or ischemic stroke from inadvertent dislodgement of calcified plaque in the carotid artery. The superior thyroid artery can communicate with the inferior thyroid artery via retrolobar and intraglandular anastomoses [11]. This anatomical architecture allows for the use of pressure-enabled delivery devices (PEDDs) to embolize the superior thyroid territory by delivering particles via accessing only the inferior thyroid artery. The unidirectional valve present within the PEDD generates a forward pressure gradient to push calibrated microspheres (100-300 μm) through intrathyroidal collaterals while preventing reflux. This approach eliminates the need to access the carotid circulation, potentially decreasing the risk of ischemic stroke [11] (Figs. 2-4).

**Figure 1.**
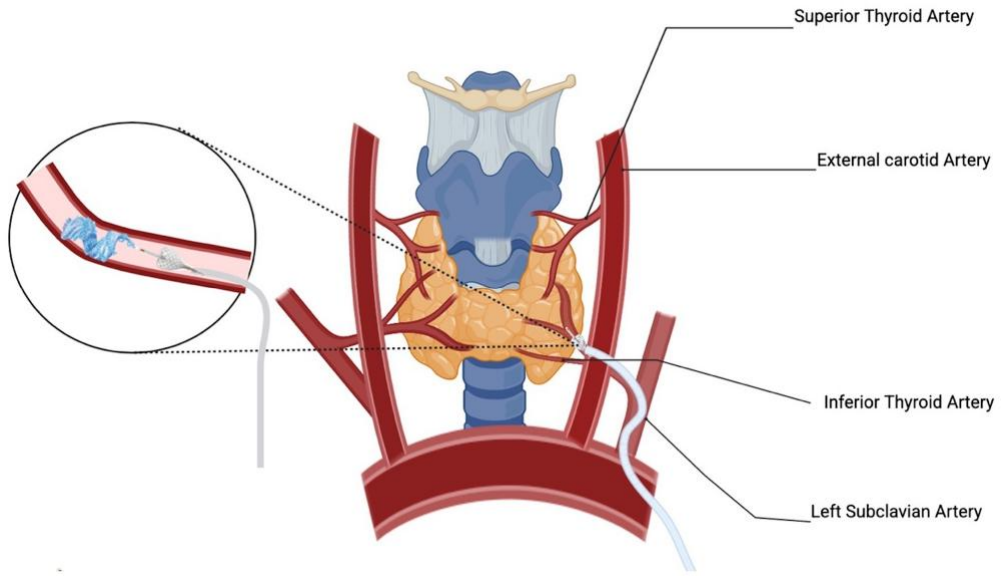
Illustration of the anatomy of the thyroid arteries and embolization of the inferior thyroid artery using a pressurized catheter.

**Figure 2.**
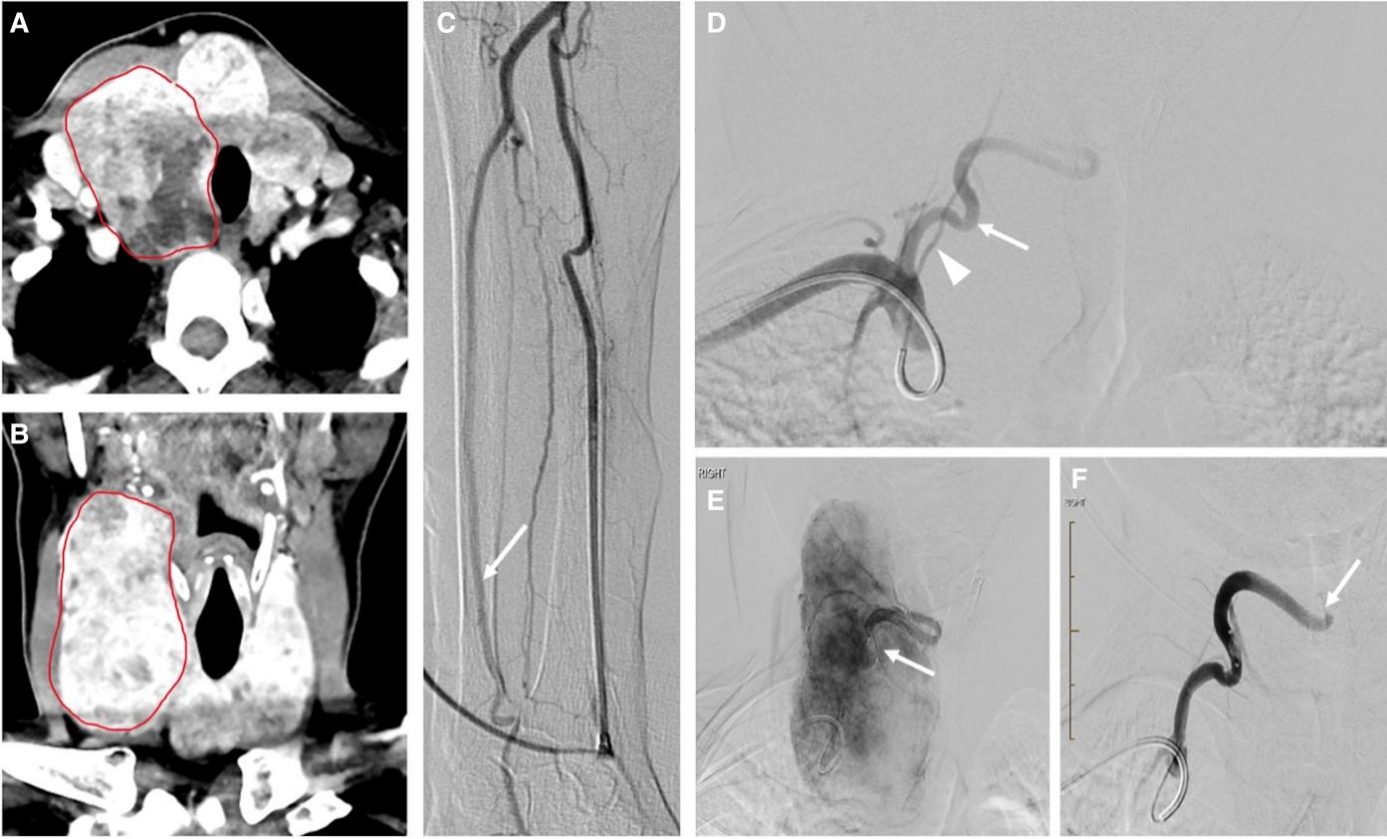
The pressure-enabled thyroid embolization technique. A, Computed tomography (CT) of the neck with contrast, axial view, demonstrating a multinodular goiter, predominantly involving the right lobe with contralateral tracheal displacement. The red circumference marks the target volume for embolization. B, CT of the neck with contrast, coronal view, demonstrating the same imaging findings. The red circumference marks the target volume for embolization. C, Right forearm angiogram demonstrates no significant radial loop and patency of the ulnar artery and palmar arch (arrow). D, Subclavian angiogram demonstrates a significantly enlarged inferior thyroid artery off the subclavian artery (arrow). Note the proximity of the ipsilateral vertebral artery (arrowhead). E, Angiography following catheterization of the right inferior thyroid artery with the pressure-enabled drug delivery device. The device is always positioned after the first turn of the artery, in its first descending segment (arrow). Note the significant goiter hypervascularity without arterial reflux. F, Flow stagnation within the right inferior thyroid artery following embolization with resolution of the parenchymal blush.

**Figure 3.**
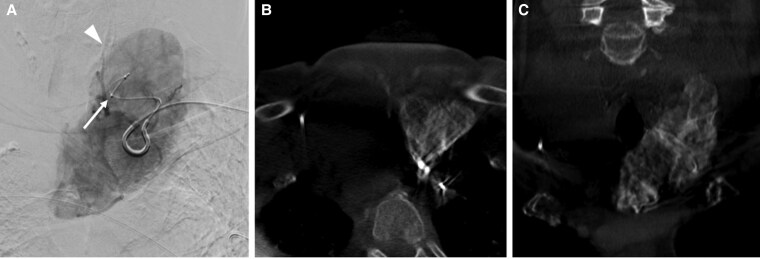
Inferior thyroid angiography with cone-beam computed tomography (CT) correlation. A, Angiography following catheterization of the inferior thyroid artery with the pressure-enabled drug delivery device. The device is always positioned after the first turn of the artery, in its first descending segment (arrow). Note the significant goiter hypervascularity without arterial reflux and opacification of the distal superior thyroid artery with flow reversal within the vessel (arrowhead). B, Axial and C, coronal cone beam CT on the angiography table demonstrating parenchymal enhancement from the catheter position for embolization without identification of vertebral or carotid branch collaterals, opacifying the whole right lobe from a single injection without catheterizing the superior thyroid artery.

**Figure 4.**
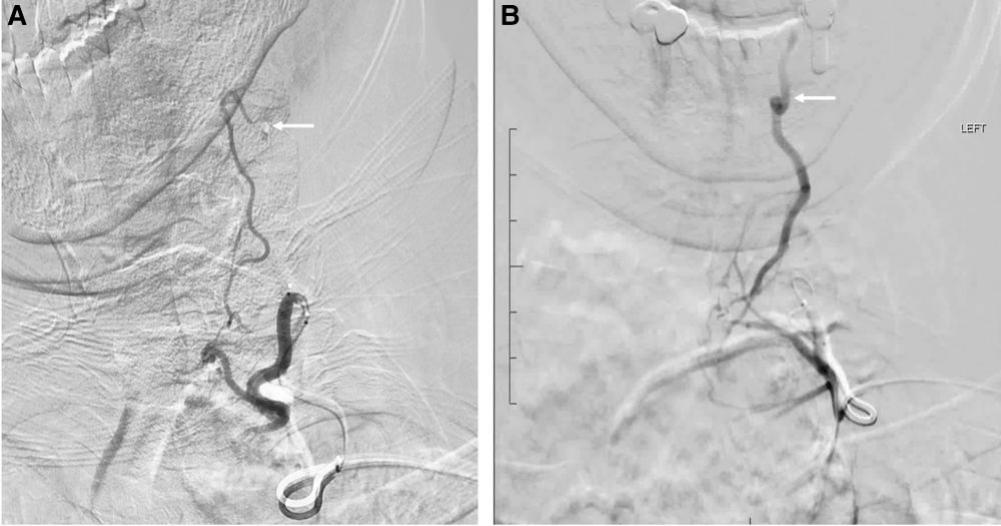
Examples of flow reversal during pressure-enabled thyroid embolization technique. A, High pressure angiography (1200 psi) following catheterization of the inferior thyroid artery with the pressure-enabled drug delivery (PEDD) device demonstrates reverse flow within the superior thyroid artery with contrast reflux into the carotid circulation (arrow). B, High-pressure angiography (1200 psi) following catheterization of the inferior thyroid artery with the PEDD device demonstrates reverse flow within a hypertrophic superior thyroid artery with retrograde opacification of the occipital artery (arrow).

The objective of this study is to evaluate the safety, feasibility, and early efficacy of pressure-enabled thyroid artery embolization (PED-TAE) via the inferior thyroid arteries in patients with symptomatic thyroid disease.

## Materials and Methods

### Patient Characteristics

Retrospective analysis of patients who underwent PED-TAE between May 2023 and July 2024. Data collection was compliant with HIPAA (Health Insurance Portability and Accountability Act) regulations and approved by the institutional review board. Embolization candidates had documented subclinical and or clinical hyperthyroidism in the presence of a toxic MNG or a toxic nodule larger than 20 mL, and/or were ineligible/refused standard-of-care therapy (surgery, radioiodine therapy, or percutaneous ablation if appropriate), presented with nonfunctioning MNGs or nodule causing compressive symptoms including but not limited to neck pain, dysphagia, stridor, exercise-induced dyspnea, and/or pressure symptoms, and had Bethesda category 2 or 3 (benign, or atypia, or follicular lesion of undetermined significance) on 2 separate fine-needle aspiration biopsy results with a benign molecular profile. Exclusion criteria included renal insufficiency, inability to tolerate angiography, including pregnancy and severe allergy to contrast media, and Bethesda 4 to 6 category on fine-needle aspiration biopsy (suspicious for follicular neoplasm, suspicious for malignancy, or malignant). The patients' demographic and baseline characteristics are presented in Table 1. Prior to embolization, all patients were examined with ultrasound and computed tomography (CT) with contrast of the neck to determine size, location, and number of the nodules. The neck CT was used to perform 3-dimensional volumetric assessment of the gland using semiautomatic imaging segmentation software (Intelerad).

**Table 1.** Patient characteristics

Characteristics	N = 22 (%)
Age, y	59.4 ± 14
Sex	
Male	7 (32%)
Female	15 (68%)
Etiology	
Multinodular goiters	11 (50%)
Toxic nodules or goiters	6 (27%)
Prethyroidectomy volume reduction	3 (14%)
Graves disease	1 (4%)
Solitary nodule	1 (4%)
Hormonal status	
Euthyroid	15 (68%)
Hyperthyroid	7 (32%)

### Thyroid Artery Embolization

The procedure was performed under moderate sedation by a single experienced (>12 years) interventional radiologist. Both femoral or radial access were performed. Patients undergoing radial access passed a Barbeau test (type D curve patients or patients with a radial artery <1.6 mm were automatically excluded from this access). A 5 Fr base catheter was used to select the inferior thyroid artery. Cone beam CT on the angiography table was then performed once the PEDD catheter was in position for embolization to ensure safety and to avoid nontarget embolization (see Fig. 3). The TriNav Infusion System was advanced coaxially through the base catheter with the valve positioned in the descending segment of the inferior thyroid artery, prior to its parenchymal bifurcation. Subsequently, the arteries were embolized with 100- to 300-μm calibrated spheres until stasis was achieved. Patients were observed following the procedure for a total period of 2 hours. The postprocedural medication included intravenous dexamethasone (8 mg) and nonsteroidal anti-inflammatory drugs to reduce the postoperative edema and inflammation (Ketorolac 15 mg IV). After discharge, patients were prescribed an oral nonsteroidal anti-inflammatory drug and a Medrol dose (methylprednisolone) pack. Thyroid hormone tests were obtained 2 weeks following the procedure to establish a new baseline and then every 3 months. A low dose of propranolol (20 mg every 12 hours) for 30 days was also prescribed and then weaned off accordingly. In patients who developed postembolization hyperthyroidism, antithyroid drugs (methimazole, propylthiouracil) and intestinal thyroid hormone binders (cholestyramine) were considered on a case-to-case basis.

### Efficacy

Technical success was defined as successful catheterization and embolization. Clinical success was measured as the absence of nontarget embolization, target volume reduction, and/or normalization of thyroid function when appropriate. Thyroid hormone tests were obtained 2 weeks post procedure to establish a new baseline and then every 3 months.

At 6 months, follow-up 3-dimensional CT volumetry was performed, and volume reduction ratio was calculated as follows:


$$\frac{\mathrm{Pre}\text{-}\mathrm{embolization}\;\mathrm{volume}\;\text{–}\;\mathrm{post}\text{-}\mathrm{embolization}\;\mathrm{volume}}{\mathrm{Pre}\;\mathrm{embolization}\;\mathrm{volume}}\times 100\text{\%}$$

Complications that occurred during and after the procedure were evaluated and categorized according to the Common Terminology Criteria for Adverse Events, version 5.0. (CTCAE v 5.0). CTCAE is a standardized tool used by clinicians to document and grade the severity of adverse events. CTCAE grades range from 0 (no adverse event) to 5 (death related to the event).

### Statistical Analyses

Continuous variables are expressed as mean and SD. Categoric variables are expressed as frequency and proportion. Thyroid volume before and after embolization were compared using a paired *t* test. All statistical analyses were performed using R software version 4.2.3 (R Foundation for Statistical Computing).

## Results

A total of 22 patients, with an average age of 59.4 ± 14 years, underwent PED-TAE using 100- to 300-μm microspheres (Embosphere Microspheres; Merit Medical). The cohort was predominantly female (68%). Most patients (82%) underwent embolization via radial arterial access.

Among the patients, 10 (45%) received preplanned unilateral embolization, while 12 (55%) underwent bilateral lobar embolization. Unilateral embolization was performed on patients with unilateral goiter or unilateral nodule. In contrast, bilateral embolization was reserved for bilateral MNGs. The underlying etiologies for treatment included 11 patients (50%) with MNGs, 6 patients (27%) with toxic nodules or goiters, 3 patients (14%) requiring prethyroidectomy volume reduction, 1 patient (4%) with Graves disease, and 1 case (4%) with a solitary nodule exceeding criteria for radiofrequency ablation (see Table 1).

### Efficacy

Technical and clinical success was achieved in all 22 patients. Based on initial angiographic findings, 34 thyroid arteries were planned for embolization, and all targeted arteries were successfully embolized. Symptomatic relief was reported as early as 2 weeks post procedure. Three patients (14%) underwent planned thyroidectomy within 72 hours after embolization as part of a neoadjuvant approach. Of the patients presenting with hyperthyroidism, 71% (n = 5) achieved a euthyroid state following the procedure. Two patients returned to their baseline hyperthyroid state during follow-up after 8 to 12 months following embolization, 1 presented with a toxic MNG, and 1 with advanced Graves disease.

### Safety Profile

Eighteen patients (82%) reported grade 1 complications classified using CTCAE v 5.0 as mild symptoms such as pain or discomfort not requiring intervention. All symptoms resolved within 2 weeks. No major complications were reported. Notably, no cerebrovascular complications were observed throughout the study. No evidence on nontarget embolization was reported.

### Gland Volume Reduction

**Figure 5.**
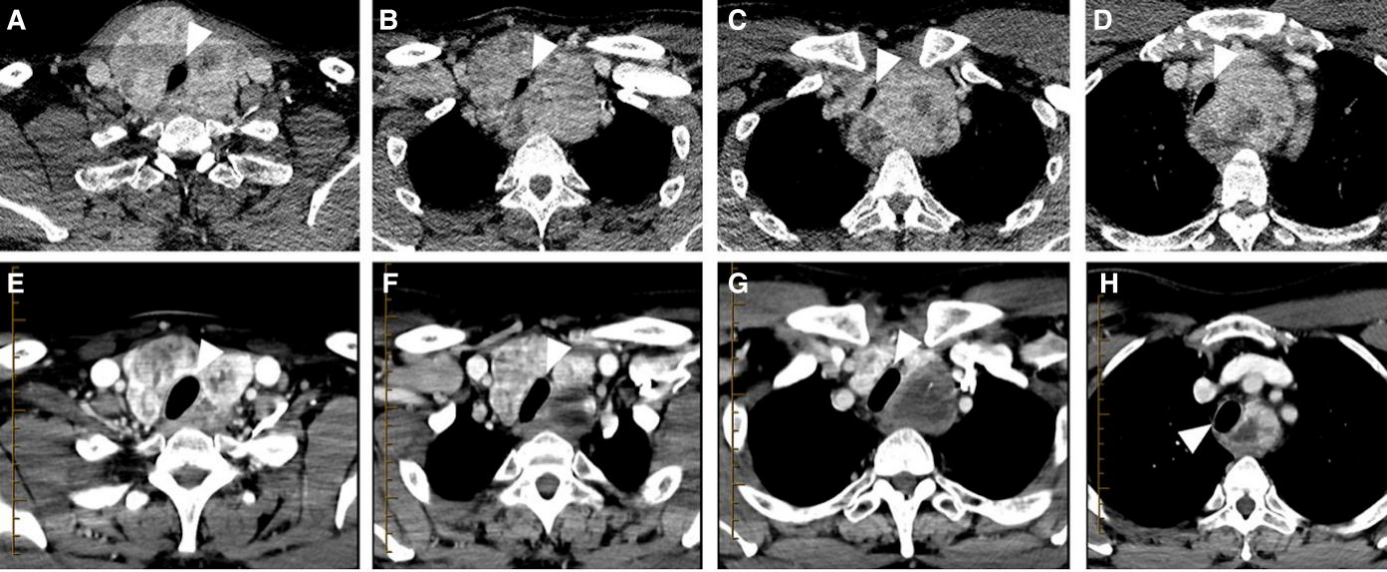
Multinodular goiter case. Preprocedural computed tomography (CT) of the neck images with contrast through the A, infraglotic supraclavicular region; B, at the level of the clavicle; C, below the clavicle; and D, in the superior mediastinum 2 cm above the carina, demonstrating a significant multinodular goiter with mediastinal extension and extreme luminal narrowing (>90%) in a 50-year-old patient presenting with dyspnea on exertion and stridor. Corresponding 3-month postprocedural CT neck images in the D, infraglotic supraclavicular region; E, at the level of the clavicle; F, below the clavicle; and G, in the superior mediastinum 2 cm above the carina demonstrate significant goiter volume reduction with reexpansion of the trachea.

**Figure 6.**
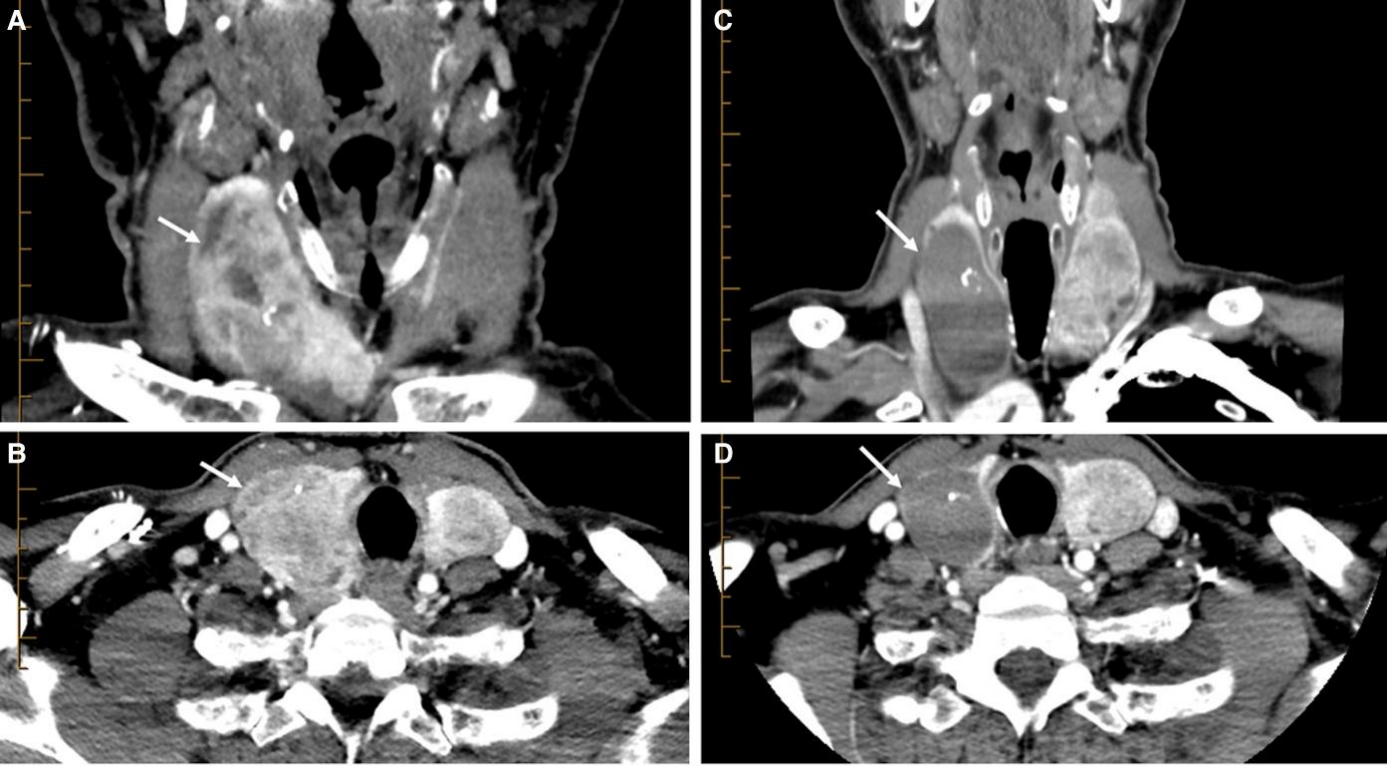
Toxic nodule case. A 68-year-old man presenting with tachycardia and anxiety secondary to hyperthyroidism. Thyroid uptake scan confirmed increased uptake in the right lobe of the thyroid. A, Coronal and B, axial computed tomography (CT) of the neck with contrast pre procedure demonstrates a significantly enlarged right thyroid lobe. Following embolization, C, coronal and D, axial CT of the neck with contrast post procedure demonstrates complete absence of enhancement of the right thyroid lobe, corresponding with glandular necrosis. This patient is currently euthyroid.

At 6 months, follow-up data were available for 18 patients. Imaging findings showed statistically significant changes in the nodules and thyroid gland (Figs. 5 and 6). Mean thyroid gland volume decreased significantly, from 184.5 ± 141.4 mL pre procedure to 49.9 ± 33.7 mL post procedure (*P* < .05). This represents a mean reduction of 73%, demonstrating the efficacy of PED-TAE in reducing gland size. One patient died due to a known history of heart disease several months after the intervention. The remaining 3 underwent thyroidectomy within 72 hours post embolization.

## Discussion

Thyroid diseases pose a considerable threat if left untreated for an extended period and are linked to higher cardiovascular disease risk, compromise of cerebral blood flow, and osteoporosis [1, 2]. Therefore, prompt treatment is warranted.

TAE, as a minimally invasive treatment, can promote apoptotic factors thus destroying the thyroid, and in turn reduces excessive thyroid hormone production [14]. Studies involving TAE in patients with Graves disease have been reported; however, data on its use in non-Graves disease conditions remain limited. A recent study evaluating the safety and efficacy of TAE in patients with nodular goiter demonstrated promising outcomes [1].

The aforementioned study achieved an average volume reduction of 56% post TAE using the traditional TAE approach, in which both the superior and inferior thyroid arteries were embolized [5]. In comparison, our patients experienced greater than 70% reduction in thyroid volume after embolizing just the inferior thyroid artery using the PEDD catheter. When using a PEDD catheter, the superior thyroid artery embolization is achieved through intrathyroidal collateral vessels, avoiding carotid access and possibly avoiding substantial complications such as ischemic stroke and nontarget embolization [11]. This allows for aggressive thyroid treatment while leaving the normal or less affected lobe fully or partially intact (see Figs. 5 and 6).

**Figure 7.**
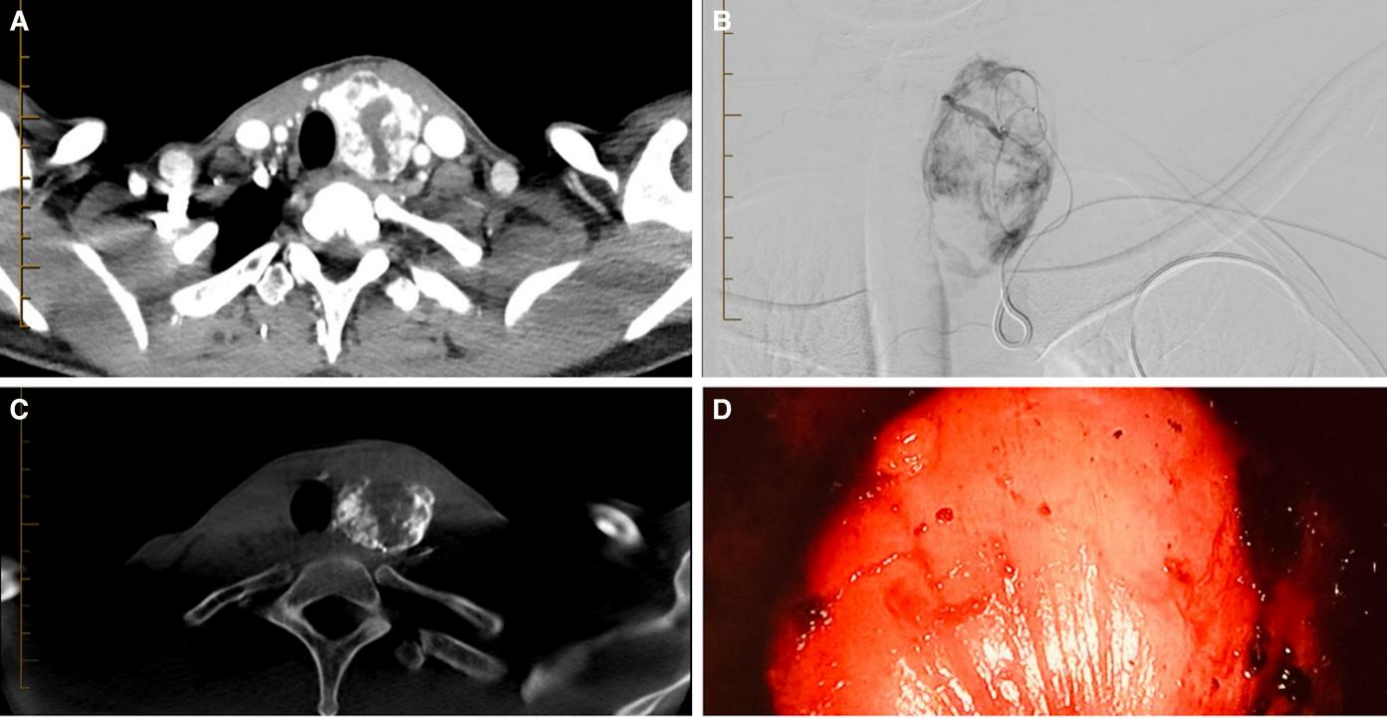
Presurgical embolization case. A 29-year-old woman presenting with a Bethesda 3 nodule measuring 20 mL in volume and associated PAX8 and PPARG mutation (70% risk of malignancy) and offered transoral thyroidectomy. A, Axial computed tomography (CT) of the neck with contrast demonstrates a hypervascular left lobe nodule. B, Angiography following catheterization of the left inferior thyroid artery with the pressure-enabled drug delivery device with the device positioned after the first turn of the artery demonstrates a significant hypervascular nodule without arterial reflux. C, Axial image obtained during intraprocedural cone beam CT acquired in the angiography table, demonstrates a peripherally hypervascular soft tissue lesion within the left lobe of the thyroid corresponding with the findings of the preprocedural CT. D, Direct endoscopic visualization of the left thyroid lobe. Note the pale appearance and the overall devascularization of the left lobe of the thyroid following embolization prior to resection.

Moreover, a subset of our patient group underwent TAE as a neoadjuvant treatment prior to thyroidectomy (n = 3). Neoadjuvant embolization is a well-known paradigm in tumor management, with proven effectiveness in reducing tumor burden in conditions such as renal cell carcinoma and spinal metastases prior to intervention [15, 16]. Our study extended this principle to thyroid volume reduction, demonstrating its potential effectiveness in managing large goiters prior to definitive treatments such as thyroidectomy (Fig. 7). It is important to mention that all patients were not candidates for other minimally invasive therapies such as ablation as there is a paucity of data regarding ablation for large thyroid nodules, and the limited studies available have demonstrated that ablation is generally ineffective for thyroid volumes exceeding 20 mL [9, 17, 18]. The average thyroid volume in the present study was 117 mL, almost 4 times above the threshold for effective ablation treatment, highlighting the challenge of using ablation as a standalone treatment modality in this patient population.

No standardized recommendations regarding the choice of embolic agent for TAE have been reported. Liquid embolic and particle embolic have been reported in previous TAE studies. Histological analysis demonstrated that the smallest diameter of the capillary network within the body of the thyroid measures around 0.04 to 0.11 mm [12, 19]. Previous recommendations suggested increasing the size of particles compared to the known size of thyroid capillaries by using 300- to 500-μm particles to minimize the risk of nontarget embolization, which is particularly relevant when embolizing the superior thyroid artery [5, 9]. In such cases, the risk of nontarget embolization arises from the complex arterial anatomy, where the superior thyroid artery branches from the external carotid artery, creating the potential for particle reflux into the carotid artery. In contrast, targeting the inferior thyroid artery, which branches from the subclavian artery, may mitigate this risk. Therefore, using 100- to 300-μm particles is technically safe considering the size of the vessels, the use of the unidirectional valved catheter, and the origin of the inferior thyroid artery, which is distal to the vertebral artery. Our study demonstrated a greater volume reduction rate compared to prior series, which we believe may be attributed to the use of smaller particles while using PED-TAE [20]. Extrapolating from preclinical and clinical liver embolization literature, pressure-enabled delivery of smaller particles may achieve a profound embolization and ischemia by overcoming the phenomenon of intratumoral (“intragoiter”) pressure, which typically impedes distal embolization [21, 22]. This pressure arises due to dense cellular proliferation within the tumor or goiter and compresses intralesional blood vessels, limiting perfusion and hindering effective particle delivery [23]. Thus, the pressure-enabled delivery generates a pressure gradient sufficient to overcome elevated intratumoral or intragoiter interstitial fluid pressure, facilitating deeper particle penetration. Moreover, the PEDD's (TriNav Infusion System) dynamic microvalve design, which centers the catheter tip within the target vessel lumen and ensures consistent infusion, further optimizes microsphere penetration within the tumor [21]. This system prevents reflux, modifies flow, and generates pressure to enable more complete particle delivery through the intrathyroid and intranodular vascular network [21]. Additionally, the collateral connections between the superior and inferior thyroid arteries enable embolic materials to reach the superior thyroid artery, which helps ensure complete embolization of the gland, further contributing to thyroid volume reduction and improved outcomes [11].

TAE in patients with Graves disease does not cause an euthyroid state. The single patient in our cohort with Graves disease did not achieve an euthyroid state, consistent with the report by Yilmaz et al [5], in which 3 of their patients with Graves disease did not achieve euthyroid status. In the present study, 7 patients had non-Graves hyperthyroidism, and 5 of 7 (71%) achieved euthyroid status, opening additional research avenues to offer PED-TAE in this scenario. None of the treated patients became hypothyroid post PED-TAE during follow-up. This reinforces the idea that PED-TAE could improve patient hormonal status in addition to decreasing nodule volume, preserving normal thyroid tissue and hence, glandular function. This is further supported by the case series by Yilmaz et al [5], in which they reported 19 of 22 (86%) effectiveness in conversion from hyperthyroidism to euthyroid status in patients with non–Graves disease.

All patients experienced symptomatic improvements as early as 2 weeks, tolerating the procedure with a low complication rate, including neck pain and subclinical hyperthyroidism, which resolved in 2 to 4 weeks conservatively. In addition, within our cohort, the feared complication of nontarget embolization of the ophthalmic artery or internal carotid branches, as well as ischemic stroke, was not observed [20]. We hypothesize that this is probably related to the novel embolization technique described, supporting the safety of the transradial approach as well as the inferior thyroid artery approach. The safety of the transradial approach for thyroid embolization in patients with large solitary thyroid nodules has been previously reported [9]. The authors reported that among 6 patients, radial artery spasms (n = 1) occurred intraoperatively, while neck pain (n = 5) and voice changes (n = 1) developed within 1 week post procedure but resolved with conservative management [9]. No major complications were observed [9]. However, the procedure was performed via the traditional TAE approach, that is, accessing the superior thyroid artery.

The present study is not without limitations; these include a small sample size and retrospective nature at a single center with a single operator, limiting the generalizability of the results. A longer follow-up duration for all patients will offer additional information in the future. Further prospective studies involving a larger number of patients should be performed to validate our observations. Hence, a multi-institutional registry study is ongoing (Pressure-enabled Retrograde Occlusive Therapy with Embolization for Control of Thyroid disease; PROTECT Registry).

### Conclusion

The present study demonstrates that PED-TAE is safe and effective for the treatment of large goiters, with a significant volume reduction of the nodule(s) and of the thyroid gland. The technique's safety profile and rapid symptom relief underscore its clinical potential in improving patients' QoL. Prospective research is warranted to establish long-term efficacy and compare outcomes directly with existing treatment modalities.

## Disclosures

S.G. and M.M. have nothing to declare. J.C.C. is a consultant for TriSalus and Pulse Biosciences and has received grant support (2018-2022 and 2025-2026) from Elesta and TriSalus, respectively. N.K. is a consultant for Boston Scientific, Sirtex Medical, Okami Medical, Terumo Medical, and Galvanize Therapeutics. R.P.T. is a senior consultant for Pulse Biosciences.

## Data Availability

Some or all data sets generated during and/or analyzed during the current study are not publicly available but are available from the corresponding author on reasonable request.

## Abbreviations

CT: computed tomography
CTCAE: Common Terminology Criteria for Adverse Events
MNG: multimodal goiter
PEDD: pressure-enabled drug delivery
PED-TAE: pressure-enabled thyroid embolization
QoL: quality of life
TAE: thyroid artery embolization
